# Impact of hyperbaric oxygenation therapy (HBOT) on renal function in human

**DOI:** 10.1038/s41598-025-10569-y

**Published:** 2025-07-11

**Authors:** Solveig Kanowski, Yuanhao Shen, Till Klein, Marcus J. Moeller, Andreas Koch, Franziska Theilig

**Affiliations:** 1 Division of Nephrology and Clinical Immunology, RWTH Aachen University Hospital, Aachen, Germany; 2 Institute of Anatomy, Christian-Albrechts-University Kiel, Otto-Hahn-Platz 8, 24118 Kiel, Germany; 3 HBO-Center Euregio Aachen, Aachen, Germany; 4 Institut für Experimentelle Medizin, Christian-Albrechts-University Kiel, Kiel, Germany

**Keywords:** Kidney, Kidney, Predictive markers

## Abstract

Hyperbaric Oxygenation Therapy (HBOT) is a widely used therapeutic option. It involves cycles with administration of 100% oxygen at increased atmospheric pressure to enhance oxygen delivery to tissues. The application of HBOT may affect all organs and tissues including kidneys which may be sensitive to the changes during HBOT. As underlying mechanisms for HBOT, the production of reactive oxygen species including superoxide, antioxidant reactions, increased plasma levels of growth factors and nuclear hypoxia-inducible factor-1α (HIF-1α) expression are discussed. Although HBOT is frequently used in man, knowledge about effects of HBOT on kidney function is still lacking in humans. The aim of this pilot study was to monitor changes in renal function parameters, including hypoxia inducible factor 1α (HIF-1α) and erythropoietin and employing urinary EV´s to document renal alterations. Test persons (*n* = 23) enrolled presented healthy renal status and received 10 HBOT sessions. Blood and urine samples were taken at the first, the fifth and the tenth HBOT session. Heart rate decreased during HBOT in male and female test persons which may be due to a stimulation of vagal nerve activity. Serum HIF-1α and erythropoietin values, blood pressure, blood and urine values for renal function parameter except for urine osmolality remained unaffected by HBOT. Urine osmolality together with the trend of renal Na^+^/K^+^/2Cl^−^-cotransporter expression on isolated urinary extracellular vesicles during HBOT significantly increased in both female in male test persons. Most likely, the generation of superoxide may account for the trend in the augmented renal NKCC2 expression and urine osmolality. HIF-1α downstream targets including renal sodium transporter affected by HIF-1α alteration remained unchanged suggesting the relative hypoxia after end of HBOT may not be sufficient. Overall, renal function upon HBOT remained largely unaffected with only minor alterations in urine osmolality.

**Supplementary Information:**

The online version contains supplementary material available at 10.1038/s41598-025-10569-y.

## Introduction

Hyperbaric Oxygenation Therapy (HBOT) is a widely used therapeutic option for a variety of clinical conditions that involves administering 100% oxygen at increased atmospheric pressure to enhance oxygen delivery to tissues. Coming from the classic Diving Medicine, HBOT is the first-line therapy in decompression sickness^1–4^, but also in barotraumatic or iatrogenic arterial gas embolism^5–7^, as well as in severe carbon monoxide-poisoning^2,8–11^. In addition, HBOT is a well-recognized additional therapeutic option in severe mixed or anaerobic infections, such as necrotizing soft tissue infections and gas gangrene^12,13^, in problematic wound therapy, such as late radiation tissue injury^14^, some forms of osteomyelitis^15,16^ or diabetic foot ulcera^17,18^ and in sudden sensorineural hearing loss or tinnitus^19–21^. Furthermore, there is growing evidence, that HBOT elicits anti-inflammatory^22–24^ and/or immunomodulatory effects^25–27^, which may offer new therapeutic options in diseases with chronic inflammation and/or inappropriate immunologic status.

HBOT comprises cycles of extreme hyperoxia and relative hypoxia after the end of an HBOT-session. The application of HBOT may affect nearly all organs and tissues and cause specific and measurable changes in laboratory parameters. HBOT with oxygen partial pressures (pO_2_) up to 300 kPa leads to a significant increase in the production of reactive oxygen species (ROS), antioxidant reactions^28^, increased plasma levels of growth factors^29^ and nuclear hypoxia-inducible factor-1α (HIF-1α) expression^30^, which all may affect body organ functions.

The kidneys receive approximately 20% of the cardiac output. At the same time, the kidneys present a heterogeneity in blood perfusion and oxygen consumption and may thus react in a sensitive way to the changes between extreme hyperoxia and relative hypoxia during HBOT. The effects of HBOT on renal function was studied in a rat model showing no alterations during 5 days HBOT treatment^31^. However, although HBOT is frequently used in man, information about the effects of HBOT on kidney function is still lacking in humans. The measurement of renal function parameters can be easily done in clinical routine. Extracellular vesicles (EV´s) excreted in the urine are predominantly derived from epithelial cells in the genitourinary system^32^. The exploration of urinary EV´s is a noninvasive method and may be used for diagnosis and prognosis of urogenital diseases since EV´s still keep the properties of the cells from which they were formed.

Thus, it was the aim of this pilot study to monitor changes in renal function parameters, including HIF-1α and erythropoietin and to use urinary EV´s to document renal alterations before and after a set of therapeutic HBOT-sessions as judged by changes in surface characteristics of urinary EVs and the abundance of renal sodium transporters in routine HBOT-treatment.

## Results

### Patient enrollment

**Fig. 1:**
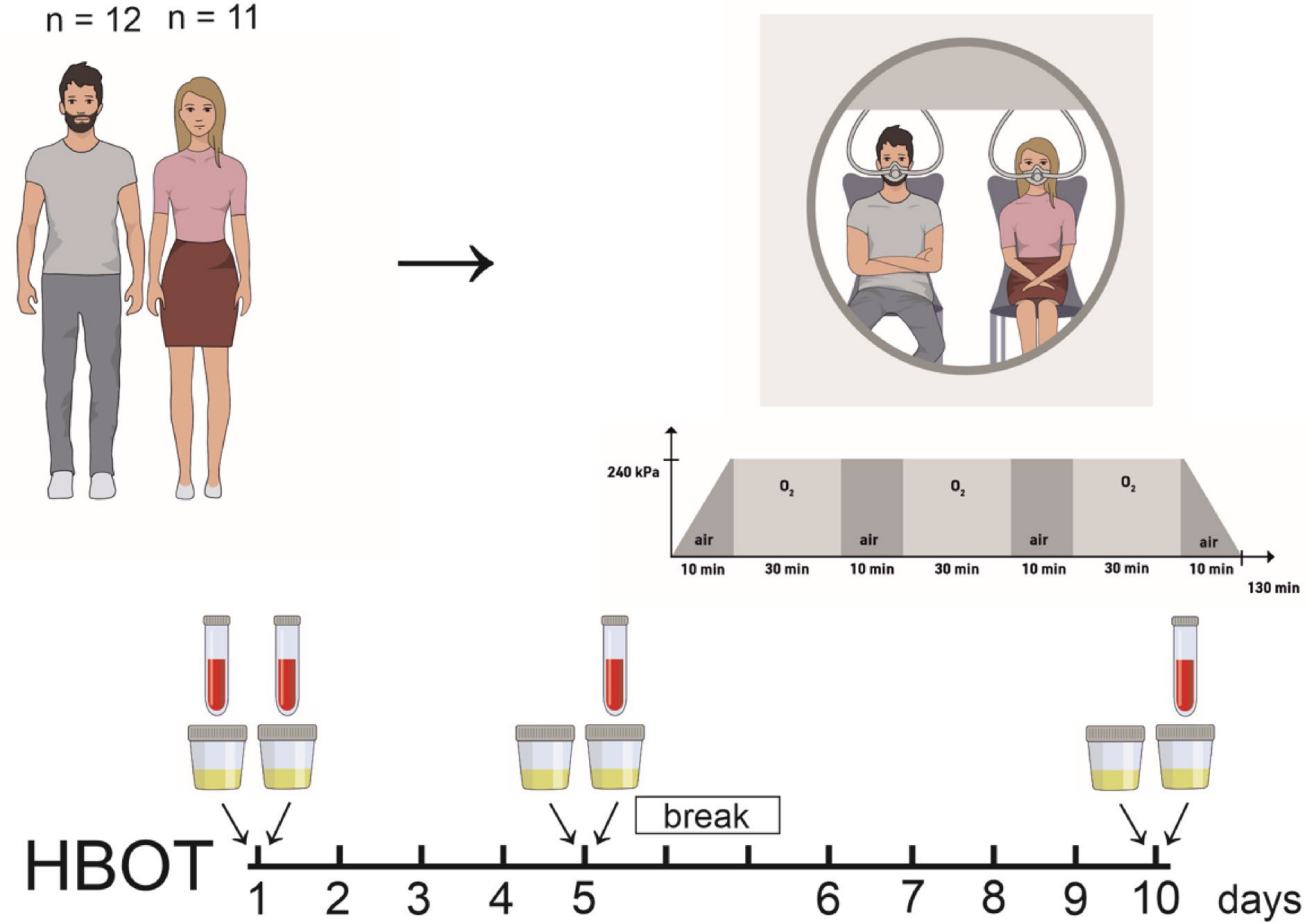
Scheme of study design. Male and female test persons were enrolled in a pilot study and blood and urine sampling was performed according the scheme.

In total *n* = 23 test persons were enrolled in this study with *n* = 12 male and *n* = 11 female test persons. The study design is illustrated in Fig. 1 showing the days of treatment performed including two days break in between.

### Cardiovascular and renal function data

**Fig. 2:**
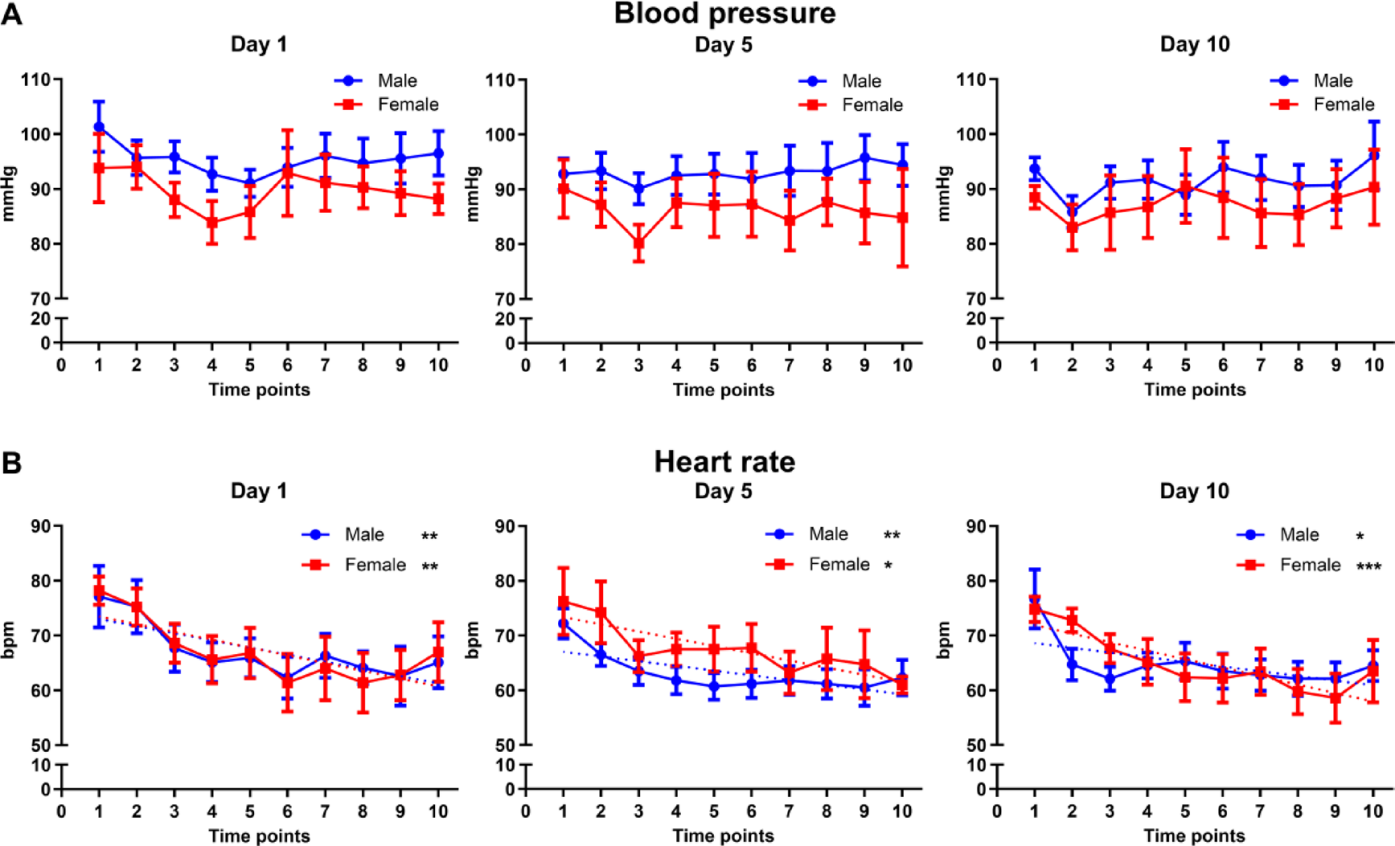
Blood pressure and heart frequency of all test persons during HBOT. (**A**) Blood pressure and (**B**) heart frequency in beats per minutes (bpm) is presented. All values are mean ± SEM, *n* = 15 with *n* = 10 male test persons and *n* = 5 female test persons. Repeated measures ANOVA followed by Tukey post hoc test, *P < 0.05; **P < 0.01, ***P < 0,001 were considered significant.

**Fig. 3:**
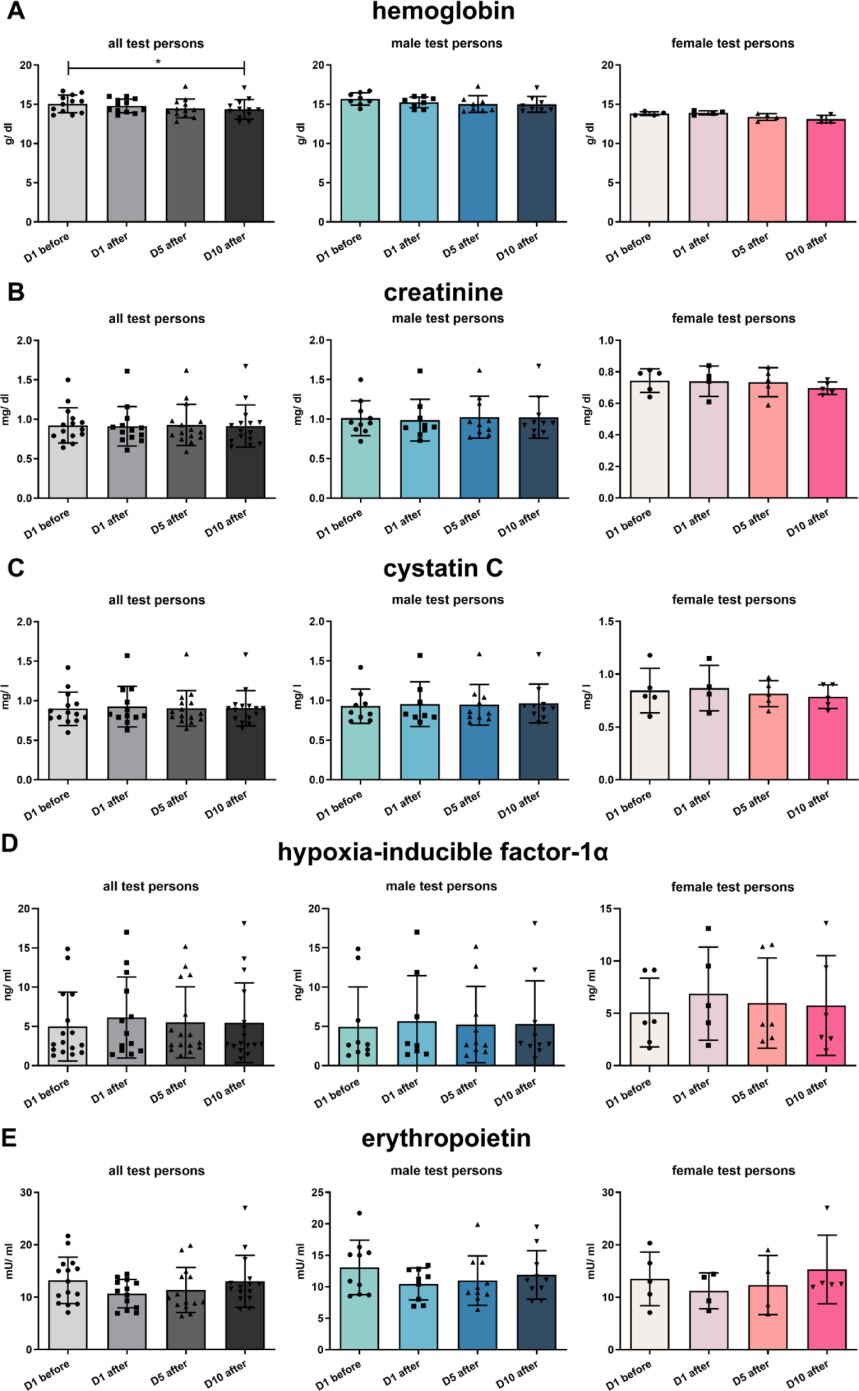
Serum parameter. Hemoglobin (**A**), creatinine (**B**), cystatin C (**C**), HIF-1α (**D**) and erythropoietin (EPO, **E**). All values are mean ± SEM, *n* = 15 with *n* = 10 male test persons and *n* = 5 female test persons. Repeated measures ANOVA followed by Tukey post hoc test, *P < 0.05 was considered significant.

Blood pressure of the test persons remained unaltered during HBOT (Fig. 2A). Heart rate of male and female test persons decreased during their stay in the hyperbaric oxygen chamber of HBOT (Fig. 2B). Hemoglobin values also decreased during the two weeks of HBOT, when comparing the first blood sampling with the tenth blood sampling of all test persons (Fig. 3A). This reduction most probably is due to a dilution effect during HBOT and not a consequence of HBO treatment. Serum creatinine, cystatin C, HIF-1α and erythropoietin (EPO) remained unaffected (Fig. 3B–E).

**Fig. 4:**
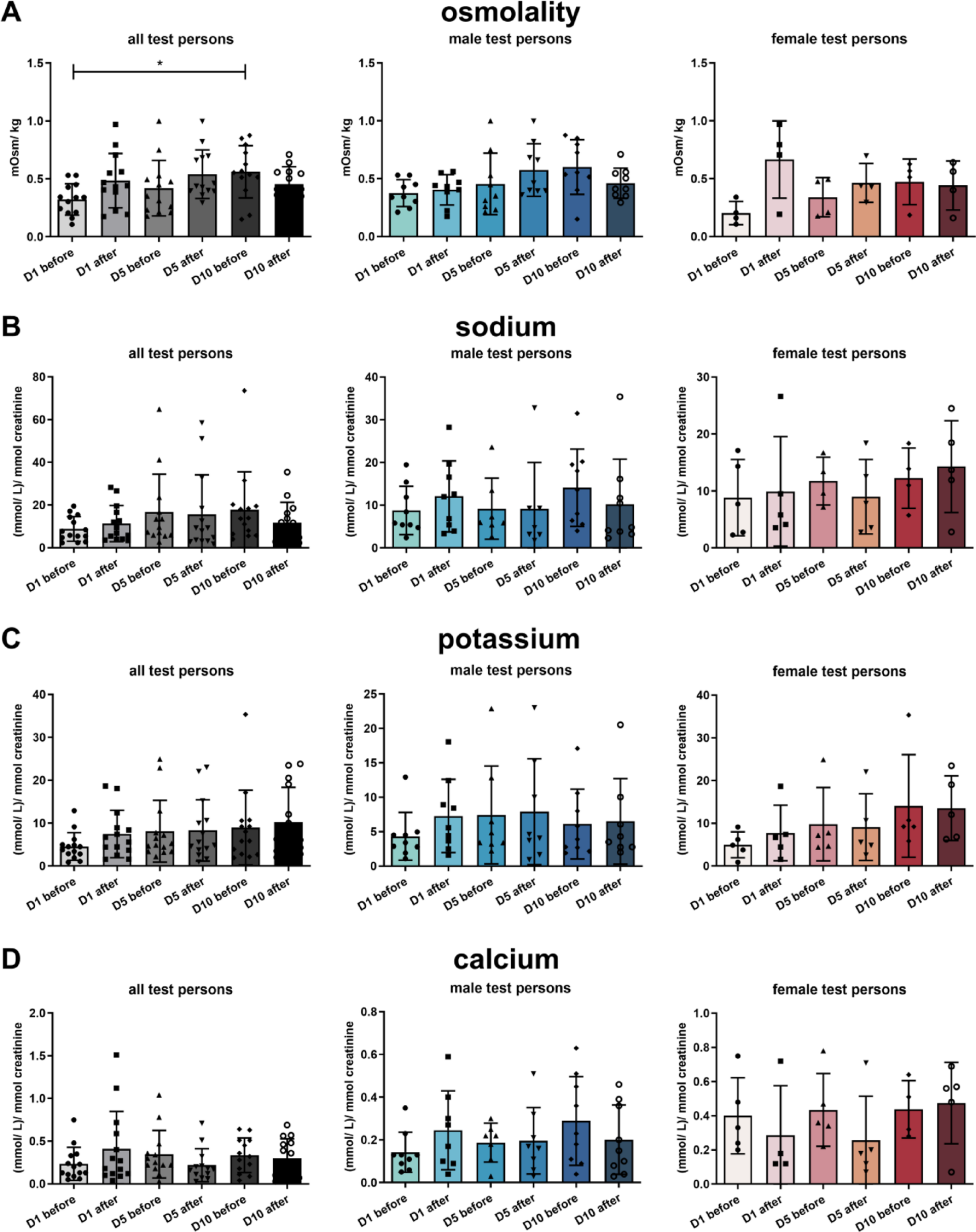
Urine parameter. Osmolality (**A**), and excretion of sodium (**B**), potassium (**C**), and calcium (**D**). All values are mean ± SEM, *n* = 15 with *n* = 10 male test persons and *n* = 5 female test persons. Repeated measures ANOVA followed by Tukey post hoc test, *P < 0.05 was considered significant.

Analysis of urine parameter revealed that urine osmolality significantly increased over the time of HBOT comparing all test persons, especially in male test persons the trend was clearly visible (Fig. 4A). The urine excretion of sodium, potassium and calcium normalized to creatinine excretion respectively, remained unaltered during the days of HBOT (Fig. 4B–D). Analysis of urine parameter obtained from control test persons remained unaltered (Supplementary Fig. 1).

### Na+/K+/2Cl–cotransporter (NKCC2) abundance increases on the surface of urinary ev´s

**Fig. 5:**
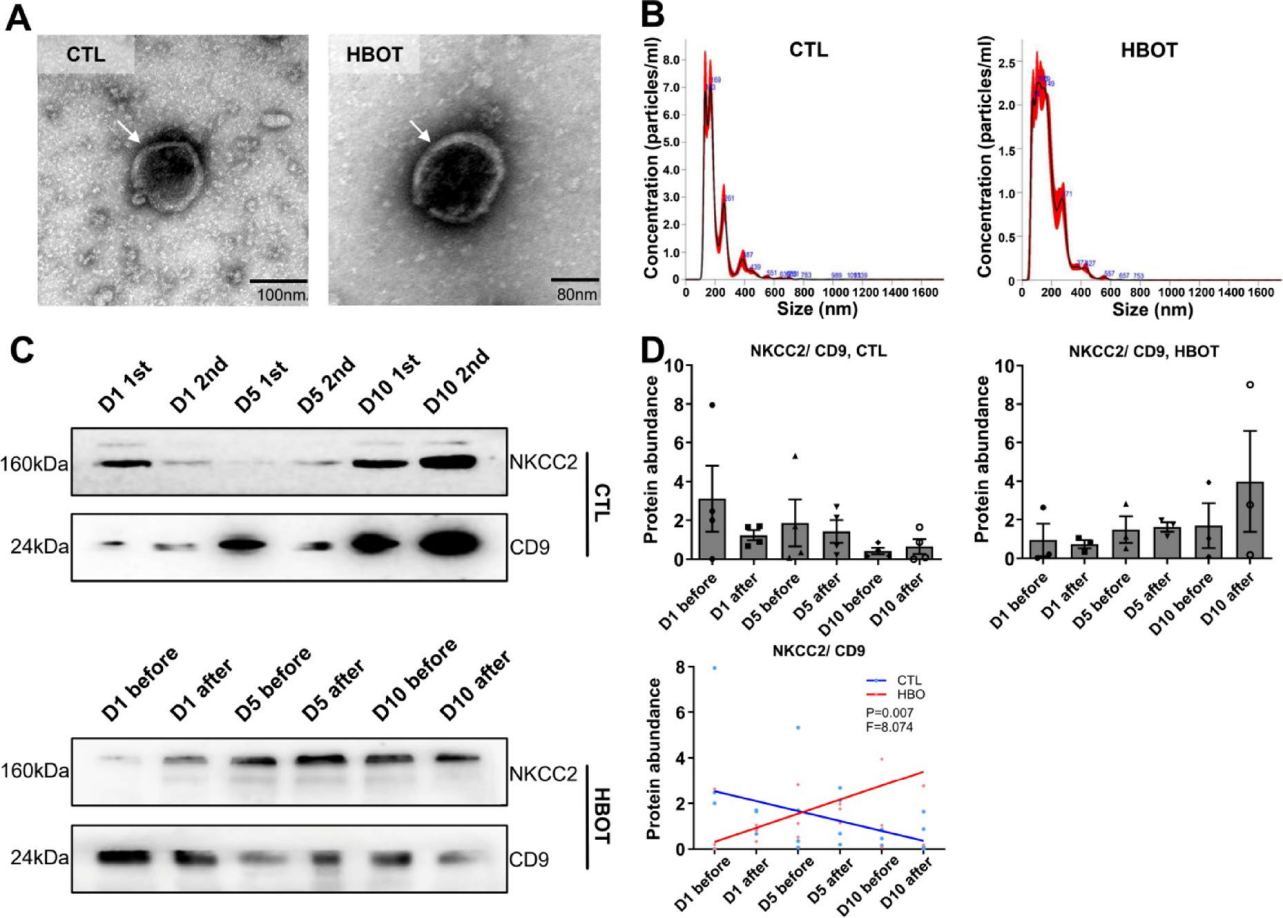
NKCC2 expression increases on urinary extracellular vesicles (EV´s). (**A**) Electron microscopy images of isolated EV showing the typical double membrane (arrow heads). (**B**) EV size distribution by Nanoparticle tracking analysis (NTA) analysis showing similar distribution between HBOT test persons and control test persons. (**C** and **D**) Representative western blots (**C**) and semiquantitative densitometric evaluation (**D**) of NKCC2 and CD9 from isolated EV´s of control test persons (CTL) and HBOT test persons (HBOT). Values are mean ± SEM, *n* = 3–4. The trend was calculated by linear regression analysis followed by slope comparation analysis, *P < 0.05 was considered significant. Original blots are presented in Supplementary Fig. 3.

Isolated urinary EV´s showed the expected size and double membrane as judged by electron microscopy (Fig. 5A). Furthermore, EV samples were controlled for proper and clean EV isolation by electron microscopy. We also determined EV size distribution by Nanoparticle tracking analysis (NTA), showing similar distribution between HBOT test persons and control test persons (Fig. 5B). HBOT with its cycles of extreme hyperoxia and relative hypoxia after the end of each HBOT-session may impact renal sodium transporter expression as demonstrated earlier^33^, therefore we tested for the abundance of renal sodium transporters and directly tested for HIF-1α downstream targets on EVs. NKCC2 abundance normalized to CD9 abundance increased over the time in HBO treated test persons compared to control test persons with a significant difference in trend line during the time (Fig. 5C and D).

Other sodium transporters such as sodium hydrogen exchanger-3 (NHE3), Na^+^/Cl^−^-cotransporter (NCC), α- and γ- subunit of the epithelial sodium channel (αENaC and γENaC) remained unchanged. Similar downstream targets of HIF-1α such as glucose transporter 1 (GLUT-1) and hexokinase-2 remained unaltered, too. All western blots and parameter analyzed are provided in Supplementary Fig. 2 and original blots are presented in Supplementary Fig. 3.

## Discussion

The kidney senses systemic oxygen, regulates erythropoiesis and hence oxygen content by hypoxia-inducible erythropoietin (EPO) expression. The characteristic of HBOT is the combination of delivering extremely high pO_2_ to the tissues, combined with in contrast to that short relative hypoxic phases during (10 min of ambient air during air-break inside an HBOT-session) and between HBOT-sessions on following days. These fluctuations in tissue oxygenation may affect renal function, which we aimed to address here.

In the presented pilot study over a period of 10 HBOT-sessions we observed in our test persons a reduction in heart rate during each HBOT, a slightly decline in hemoglobin, an increased urine osmolality together with increased NKCC2 surface EV over ten HBOT-sessions.

The observed reduction in heart rate was described previously^34^. Reduced heart frequency together with increased blood pressure during HBOT were also described as rare potential serious consequences of HBOT^35^. Therefore, authors suggested targeted cardiac investigation prior to HBOT to diagnose patients with high-risk features. HBOT was shown to stimulate vagal nerve activity with sinus bradycardia^34^. As underlying mechanism of the vagal nerve activation, a stimulation of neurons in the vagal dorsal motor nucleus and nucleus solitarius was identified.

We further observed slightly reduced hemoglobin values over time, but only, when the first blood sample, which was taken before the first HBOT, was compared to the other samples, which were all taken after the HBOT sessions. One factor, that may have contributed to some decrease in hemoglobin is the overall amount of blood taken for clinical analysis from the patients, which was in sum about 50–70 ml (see Fig. 1). And, since the hemoglobin values of the blood samples taken after the HBOT sessions differed not significantly, the 500 ml of fluid intake, that was provided during each therapy session, may have caused an additional plasma dilution effect. This fits to current literature, since alterations in hemoglobin during an HBOT series have not been described yet. We also determined serum HIF-1α and erythropoietin levels in our test persons throughout the two weeks of HBOT. HIF-1α and EPO values remained in a normal physiological range with only minor alterations. During hypoxia, HIF-1α expression strongly increases in renal epithelial cells^36,37^, whereas EPO is mainly produced and released by renal cortical interstitial fibroblast in a HIF-2α -dependent manner^38,39^. These results suggest that the fluctuation in tissue oxygenation during HBOT is not sufficient to induce tissue hypoxia with subsequent HIF-1α stabilization and EPO release that is detectable in serum. Although, HIF-1α alterations during HBOT were described in humans and in animal models^40,41^ the difference to our study may be related to the duration of HBOT. In our study, the HBOT of the enrolled patients was shorter (two cycles of 5 days) than other studies of HBOT in human^21,41^ and may not have led to significant HIF-1/2α stabilization yet.

The analysis of the urine parameters revealed significantly increased urine osmolality which in general is affected by the amount of sodium excreted. In addition, hypoxia was shown to affect renal sodium transporter expression^33,42^ we sought to address their expression levels by analyzing excreted urinary vesicles. In congruence with increased osmolality, we observed higher NKCC2 expression during HBOT. Since we did not find an indication of tissue hypoxia in our study, another explanation for the higher NKCC2 expression could be the HBOT-induced superoxide generation, which we have shown to occur in HBOT recently^43^. Thus, superoxide was shown to play a role in renal sodium transport^44^ and was nicely demonstrated to enhance surface NKCC2 expression in the thick ascending limb possibly explaining the rise in urine osmolality over time during HBOT^42^. Other renal sodium transporter such as NHE-3, NCC, αENaC and γENaC remained unaltered. Those transporters were shown to be affected by manipulation of renal HIF-1α expression^33^, suggesting that the relative hypoxia occurring during (the 10 min air-breaks every 30 min) and between the HBOT-sessions may not be sufficient to provoke changes. This is also in agreement with HIF-1α and erythropoietin serum levels which remained unaltered. In addition, we tested partially for GLUT-1 and hexokinase-2 expression on the urine EV fractions. Both proteins are known downstream targets of HIF-1α. Again, no alterations or even trends were observed supporting the idea that only very low or short occurrence of relative hypoxia may take place after HBOT.

The limitation of our study is the n-number of test persons enrolled and the duration of HBOT. Since the enrolled patients had to be free of any renal dysfunction, our study-cohort consisted of patients with different forms of subacute hearing problems, where a therapeutic attempt with HBO is indicated, but with a limited number of HBO-sessions, normally about ten. Future studies with longer duration of HBOT would be helpful to demonstrate whether NKCC2 augmentation persists.

## Conclusion

HBOT in humans affects heart frequency during the HBOT-session and over time it may increase renal NKCC2 expression and urine osmolality. The short phases of relative hypoxia during HBOT and the return of pO_2_ from hyperoxia to normoxia after HBOT may have only initiated effects to a minor extent in this study without altering HIF-1α, EPO or HIF-1α downstream targets. Overall, renal function remained largely unaffected.

## Methods

### Study design

Patients enrolled in this study were diagnosed for sudden sensorineural hearing loss or tinnitus, they showed a healthy renal status and presented themselves for hyperbaric oxygen therapy (HBOT). In total *n* = 23 test persons, ageing between 25 and 70 years, were enrolled in this study with *n* = 12 male and *n* = 11 female test persons. Among the test persons *n* = 10 male and *n* = 5 female test persons underwent HBOT and *n* = 2 male and *n* = 6 female test persons followed the same protocol without receiving HBOT. 10 HBOT sessions were applied on five days a week with weekend-break, following a regular clinical schedule (100% O_2_ at 240 kPa for 90 min with two 10 min air-breaks, over all 130 min). Each HBOT session started at the same time in the morning.

Blood samples were taken before and after the first, after the fifth and after the tenth HBOT session. Urine samples were collected before and after the first, the fifth and the tenth HBO treatment. Blood pressure and heart rate were monitored every 15 min during the 130 min stay inside the hyperbaric oxygenation chamber of the first, the fifth and the tenth HBO treatment. During HBOT patients received 500 ml drinking water.

This study (named RENOX) was approved by the local Ethic committee of the medical association Aachen, Germany under the number 2,021,469. All experiments were performed in accordance with the guidelines and regulations of the Ethic committee. All participants and/or their legal guardians provided written informed consent.

### Blood and urine collection and analysis

Blood sample analysis was performed in a routine medical laboratory (MVZ Stein, Mönchengladbach, Germany). Hemoglobulin (Hb in g/dl) was determined by photometry, cystatine C (cysC in mg/l) by turbidometry, creatinine by Jaffé photometry, HIF-1α were determined by ELISA (A313500, VWR) and erythropoietin was measured in the medical laboratory (MVZ Stein, Mönchengladbach, Germany). Urine samples were taken before and after the first, the fifth and the tenth HBOT and treated with protease inhibitor phenylmethylsulfonyl fluoride (PMSF, 0.1 mM, Thermofisher, Germany). Electrolyte concentration in urine and serum samples were determined by flame photometry (EFOX 5053, Eppendorf, Hamburg, Germany) and urine osmolality by algoskopy (Osmomat 3000, Gonotec, Berlin, Germany). Serum and urine creatinine were determined by colorimetric quantitative determination of creatinine (PAP test, LT-CR0106, Labor & Technik, Berlin, Germany).

### Urinary extracellular vesicle isolation

Urinary extracellular vesicles (EV) were isolated according the improved protocol using the differential ultracentrifugation approach including DTT-mediated recovery of Tamm Horsfall protein (THP)-entrapped EVs and alkaline washing^45^. All urine samples of one patient (100 ml) were thawed at 4 °C and normalized to the lowest creatinine value received per sample of the patient. Samples with higher creatinine value were adapted to equal volume using PBS. Normalized and equalized urine samples were transferred to 50 ml falcon tubes (Sarstedt, Germany) and centrifuged at 20,000 g (Minifuge 1-SR, Kendro, Germany) for 30 min to remove cells and cell debris. The supernatant was kept on ice. The resulting pellet was resuspended in isolation buffer containing 0.25 M sucrose, 10 mM HEPES, 1 mM EDTA, pH 7.4 with DTT at a final concentration of 200 mg/ml DTT and after incubation for 5 min, centrifuged at 20,000 g for 30 min. The resulting supernatant was pooled with the first supernatant and centrifuged at 175,000 g (L7-65, Beckman) for 2 h. Resulting pellet was resuspended in 5 ml alkaline solution (50 mM Na2Co3, pH = 11) and was passed through a 0.22 µM PES syringe filter (Millipore SLGP033RS) to remove large particles. The filter was rinsed with an additional 5 ml of alkaline solution and total filtrate was ultracentrifuged at 175,000 g for 2 h and final pellet resuspended in 200 µl PBS and used for western blotting.

### Transmission electron microscopy (TEM)

Aliquots of the resuspended EV pellets (P4) in PBS were further diluted in PBS and a small volume of the diluted EVs were transferred to negative-glow discharged continuous carbon grids (Science Service, Munich, Germany) and stained with a half‐saturated solution of uranyl acetate. The images were generated using a JEOL‐1400‐Plus‐TEM (JEOL Germany, München) with integrated TemCam‐F416 camera (TVIPS, München).

### Nanoparticle tracking analysis (NTA)

The EV size distribution and concentration of the particles in the resuspended EV pellet were determined via NTA with a NanoSight NS300 instrument (Malvern, Panalytical, Malvern, UK) using NanoSight software version 3.40 (Malvern). Samples were diluted in PBS to achieve a particle concentration within the optimal range for analysis reported by the manufacturer.

### SDS-Page and Western blot

Proteins were solubilized and SDS gel electrophoresis was performed on 10% polyacrylamide gels. After electrophoretic transfer of the proteins to nitrocellulose membranes, equity in protein loading and blotting was verified by membrane staining using 0.1% Ponceau red. Membranes were probed with primary antibodies (s. list) and then exposed to HRP-conjugated secondary antibodies (Dianova, Hamburg, Germany). Immunoreactive bands were detected by chemiluminescence using Immobilon Western HRP substrate (Millipore, Darmstadt, Germany) in combination with the chemiluminescence imaging system Fusion SL (Peqlab, Erlangen, Germany) and further analyzed using ImageJ software.

### Antibodies

The following antibodies were used: rabbit anti-NHE3 (Novus Biologicals, NBP1-82574), guinea anti-NKCC2 (gift. S. Bachmann), sheep anti-NCC (S961B, University of Dundee), rabbit anti-αENaC (StressMarq, SPC-403D), rabbit anti-γENaC (StressMarq, SPC-405D), rabbit anti-GLUT1 (Merck Millipore, 07-1401), rabbit anti-NHE3 (NBP1-8257 Novus), anti-CD9 (invitrogen, MA5-31980), and rabbit anti-hexokinase-2 (Poteintech, 22029-1-AP).

### Statistical analysis

Statistical analysis was performed using GraphPad Prism 7.0 (GraphPad Prism). Data are provided as arithmetic means ± SEM with n representing the number of used samples. To test statistical significance, repeated measures ANOVA followed by Tukey post hoc test and linear regression analysis followed by slope comparation analysis was performed. *P < 0.05, **P < 0.01, ***P < 0.001 were considered statistically significant.

## Electronic supplementary material

Below is the link to the electronic supplementary material.

Supplementary Material 1

## Data Availability

The data that support the findings of this study are available in the Materials and Methods, Results, and/or Supplementary Material of this article.
