# Supplementary material for: Impact of hyperbaric oxygenation therapy (HBOT) on renal function in human
Source: Sci Rep. 2025 Jul 11;15:25143. doi: 10.1038/s41598-025-10569-y (PMC12254248; doi:10.1038/s41598-025-10569-y)
Supplement: Supplementary file 1 — Supplementary Material 1 [file 41598_2025_10569_MOESM1_ESM.pdf]

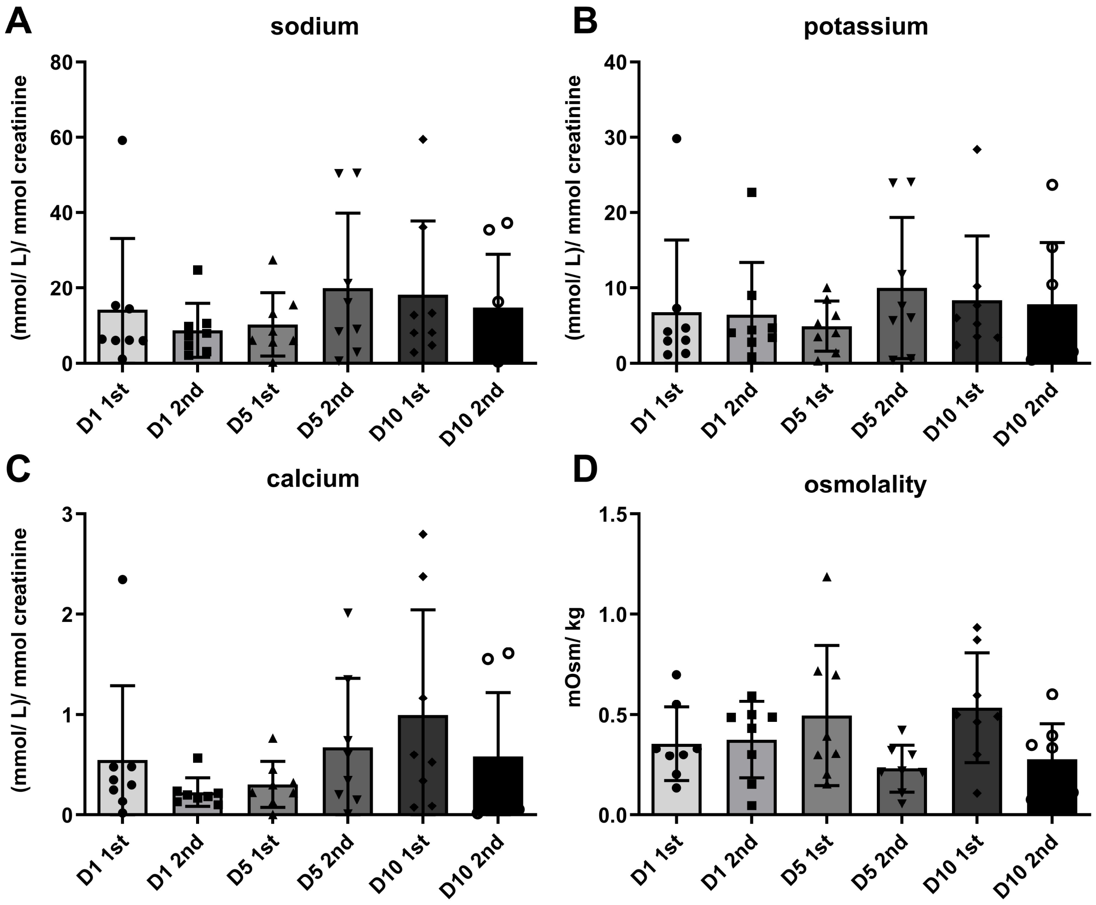

Supplementary Figure 1. Urine parameter of control test persons. The excretion of sodium (A), potassium (B), calcium (C), and osmolality (D). All values are mean ± SEM, n = 8 with n = 2 male test persons and n = 6 female test persons. Repeated measures ANOVA followed by Tukey post hoc test was performed.

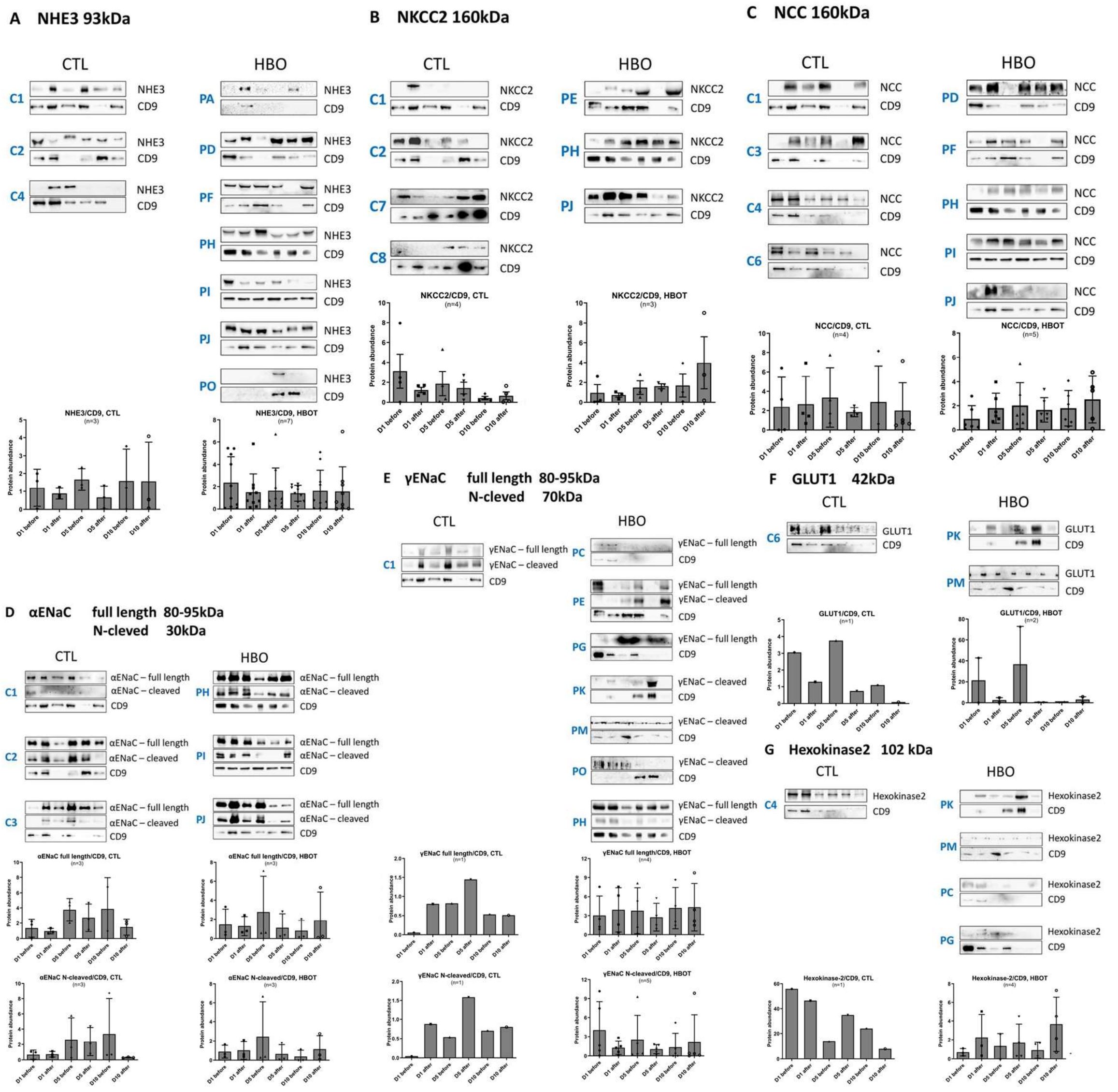

Supplementary Figure 2. Summary of western blots and densitometric evaluation of urinary surface EV expression. Among the tested parameter were sodium hydrogen exchanger-3 (NHE3, **A**), Na$^+$/K$^+$/2Cl$^-$-cotransporter (NKCC2, **B**), Na$^+$/Cl$^-$-cotransporter (NCC, **C**), α-subunit and γ-subunit of epithelial sodium channel (αENaC, **D**; γENaC, **E**), glucose transporter-1 (GLUT-1, **F**) and hexokinase-2(**G**).

Supplementary Figure 3. Original blots of protein abundance of urinary extracellular vesicles

Control 1

αENaC

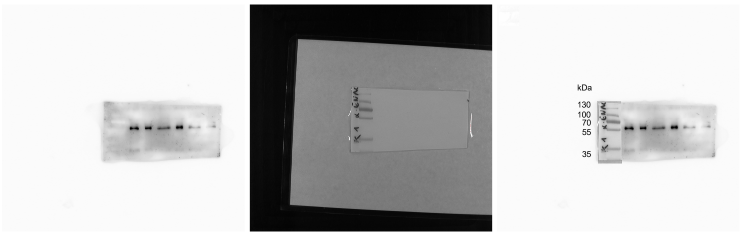

detection marker merge

γENaC

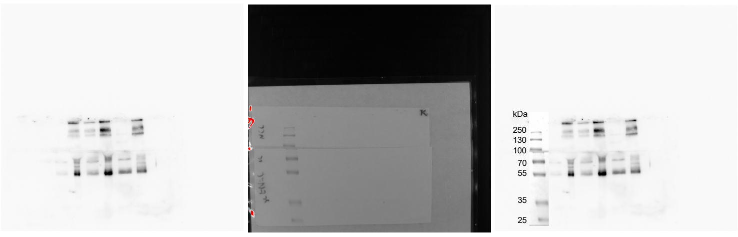

detection marker merge

CD9

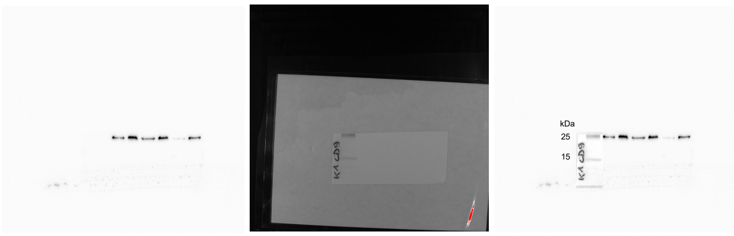

detection marker merge

NCC

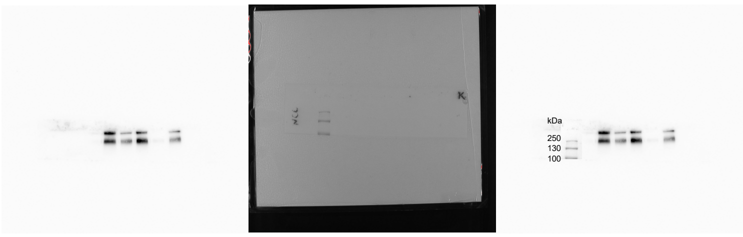

detection marker merge

NHE3

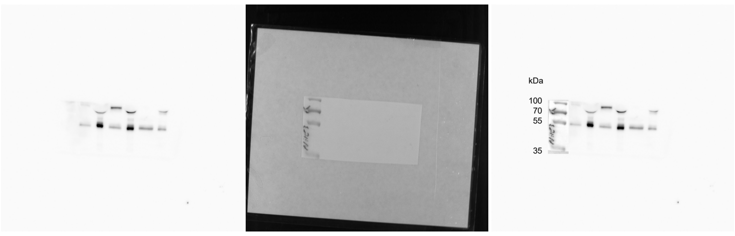

detection marker merge

NKCC2

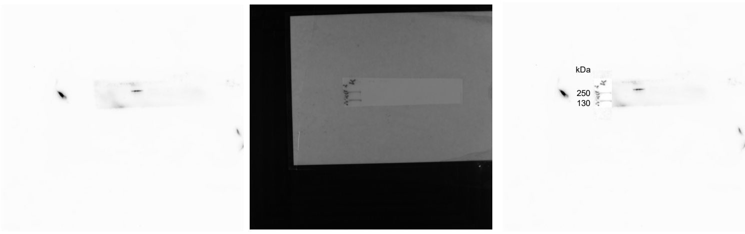

detection marker merge

Control 2

αENaC

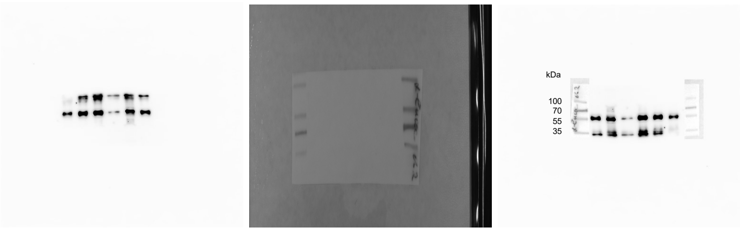

detection marker merge

CD9

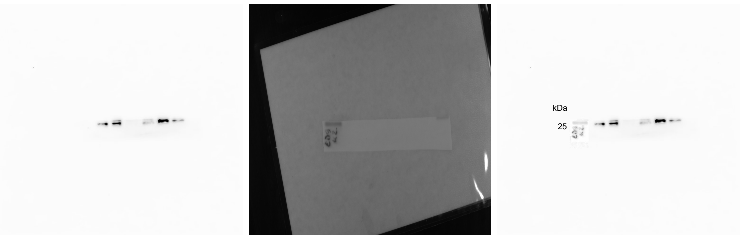

detection marker merge

NHE3

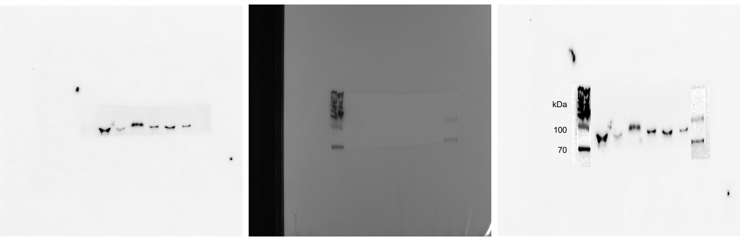

detection marker merge

NKCC2

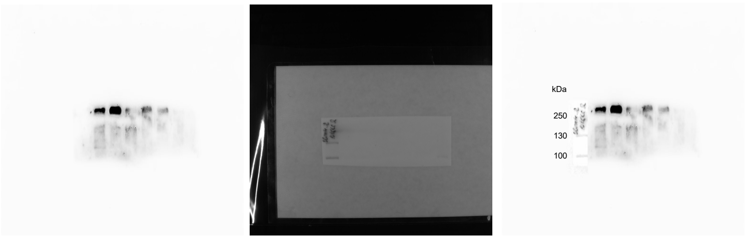

detection marker merge

Control 3

αENaC

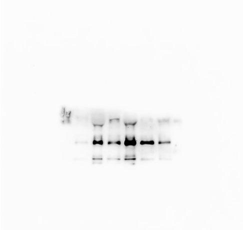

detection

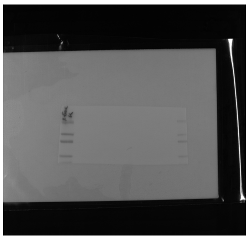

marker

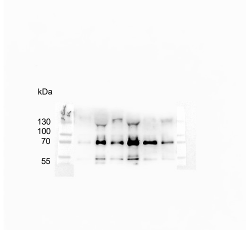

merge

CD9

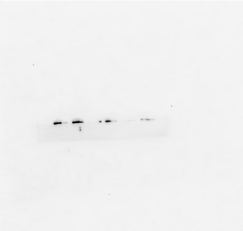

detection

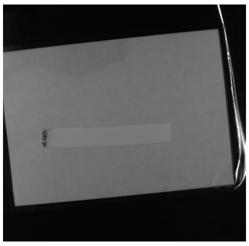

marker

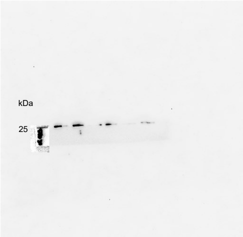

merge

NCC

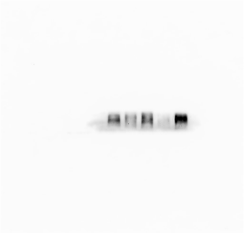

detection

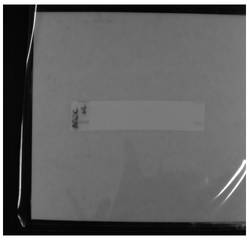

marker

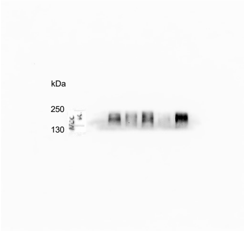

merge

Control 4

NCC

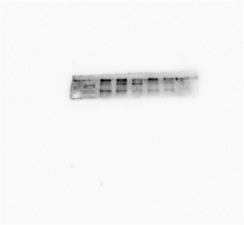

detection

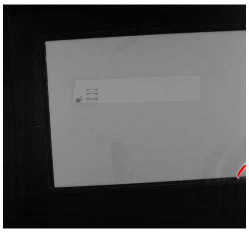

marker

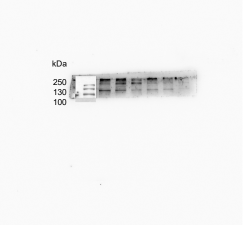

merge

CD9

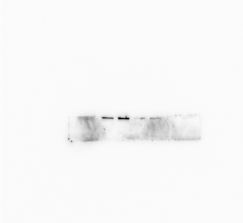

detection

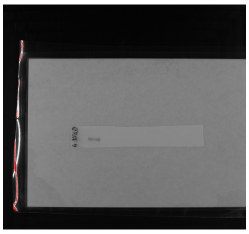

marker

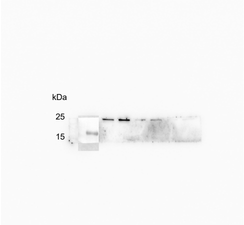

merge

NHE3

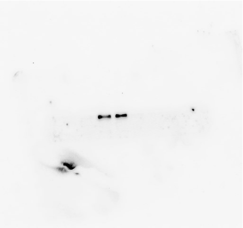

detection

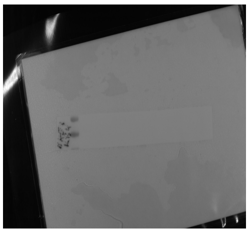

marker

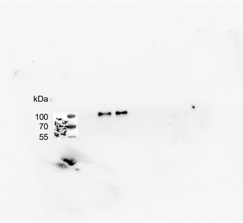

merge

Hexokinase 2

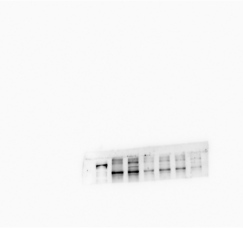

detection

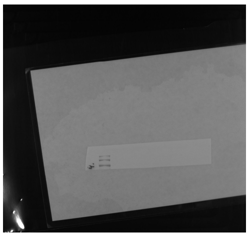

marker

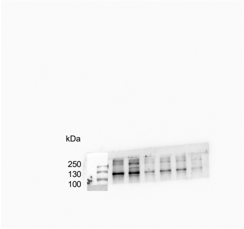

merge

Control 6

NCC

detection

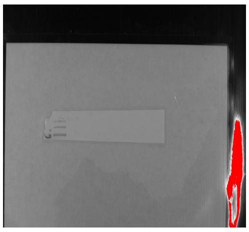
marker

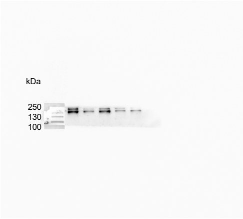

merge

CD9

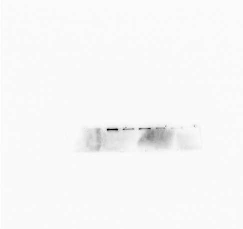
detection

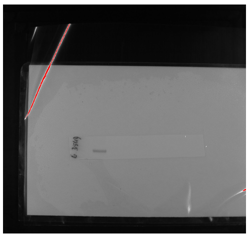
marker

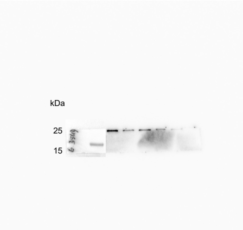

merge

GLUT1

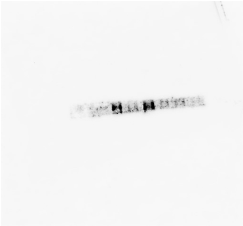
detection

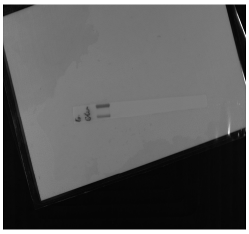
marker

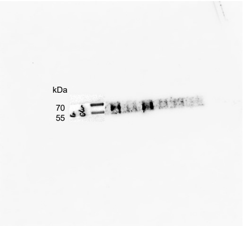

merge

Control 7

CD9

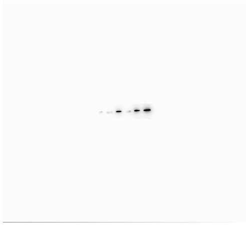

detection

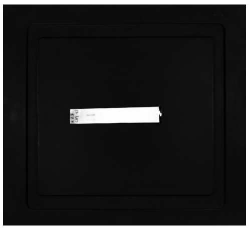

marker

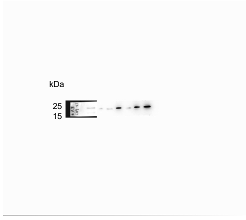

merge

NKCC2

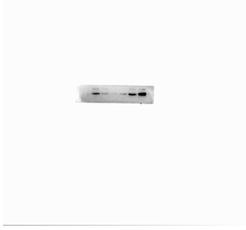

detection

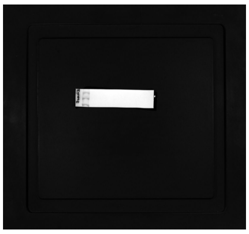

marker

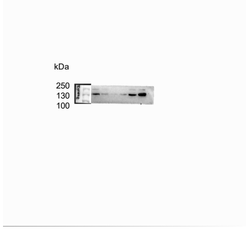

merge

Control 8

CD9

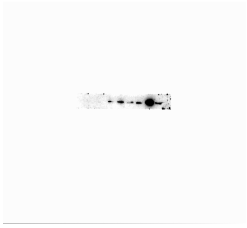

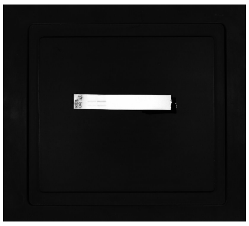

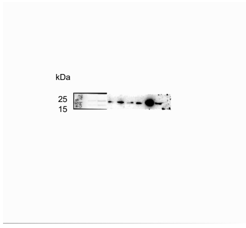

detection marker merge

NKCC2

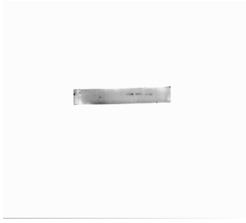

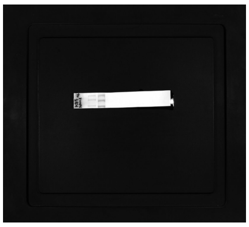

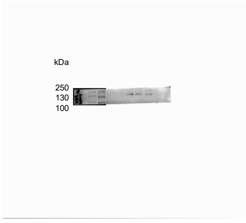

detection marker merge

Patient A

CD9

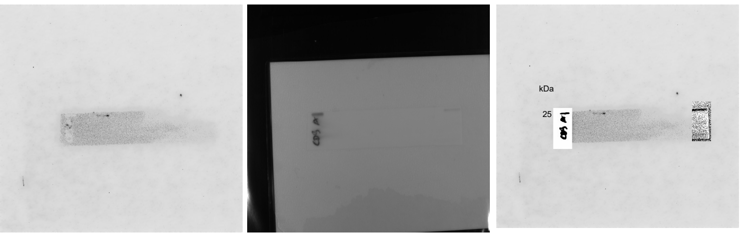

detection marker merge

NHE3

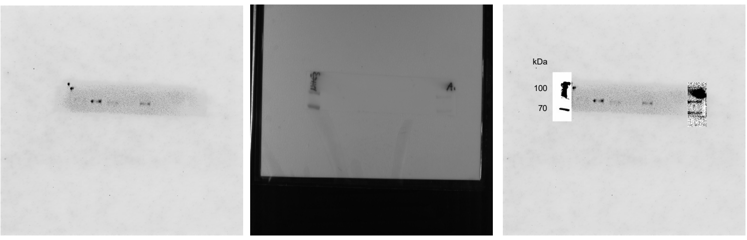

detection marker merge

Patient C

CD9

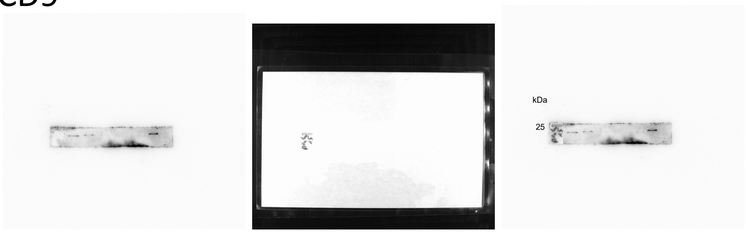

detection marker merge

γENaC

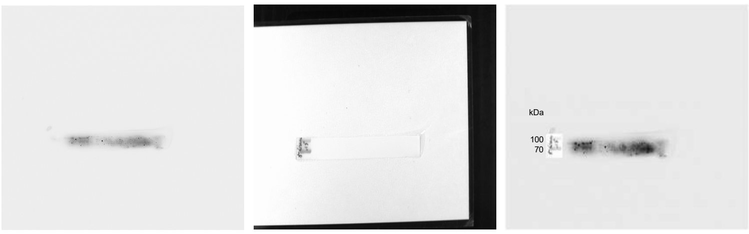

detection marker merge

Hexokinase 2

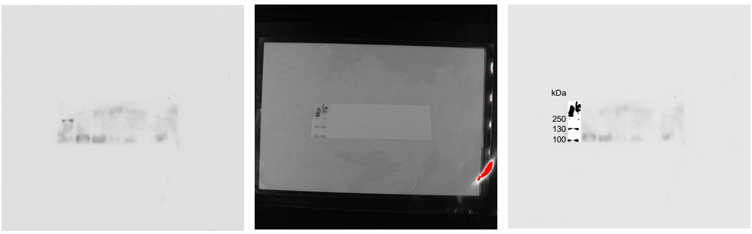

detection marker merge

Patient D

NCC

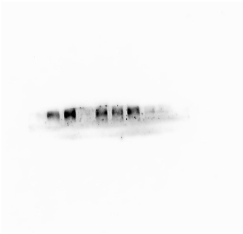

detection

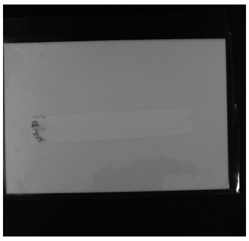

marker

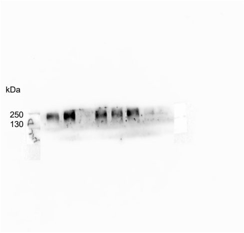

merge

CD9

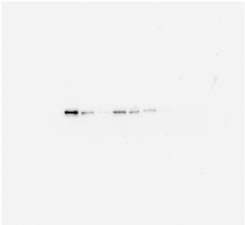

detection

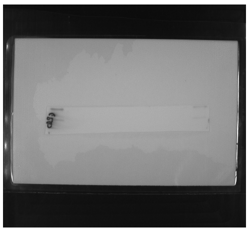

marker

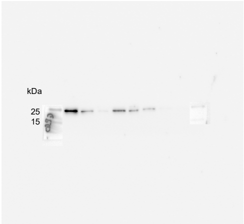

merge

NHE3

detection

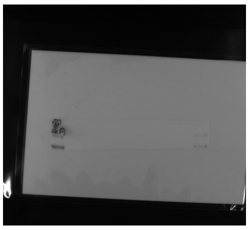

marker

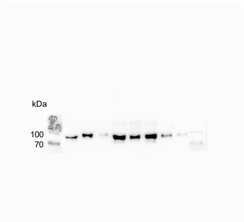

merge

Patient E

γENaC

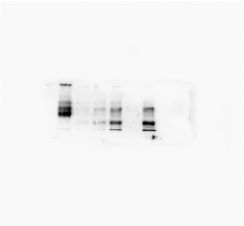

detection

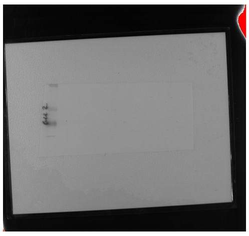

marker

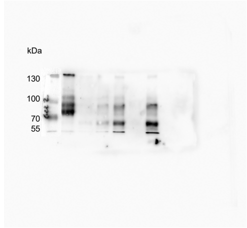

merge

CD9

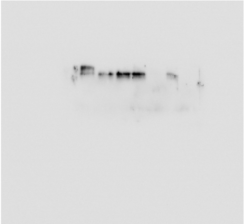

detection

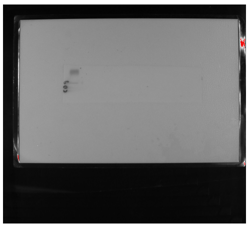

marker

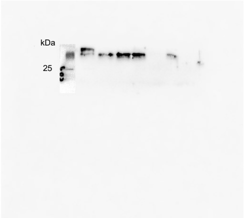

merge

NKCC2

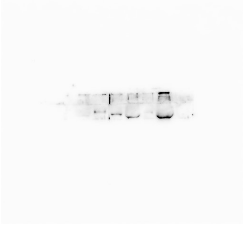

detection

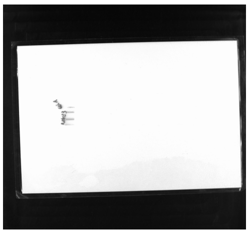

marker

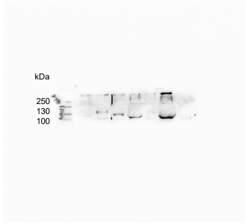

merge

Patient F

NCC

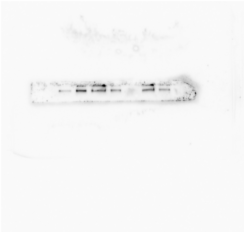

detection

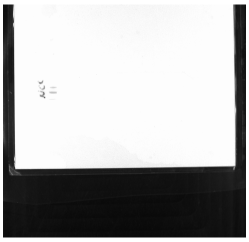

marker

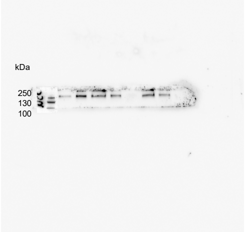

merge

CD9

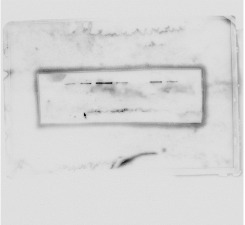

detection

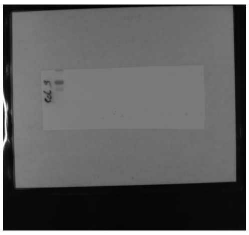

marker

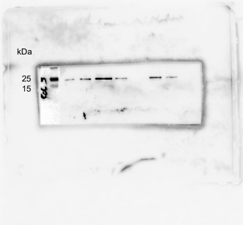

merge

NHE3

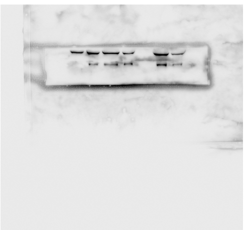

detection

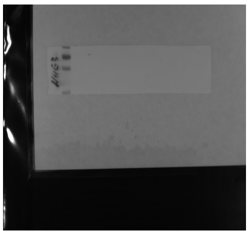

marker

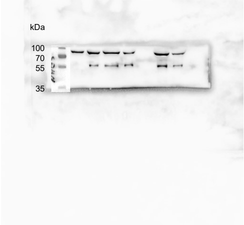

merge

Patient G

CD9

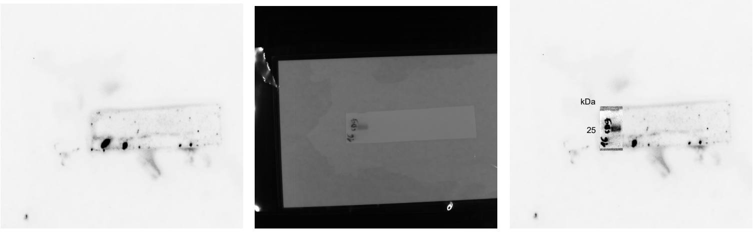

detection marker merge

Hexokinase 2

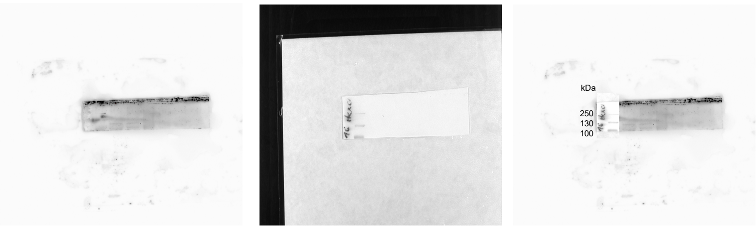

detection marker merge

Patient H

αENaC

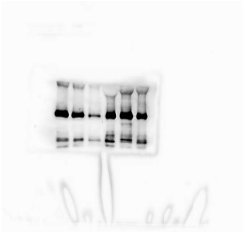
detection

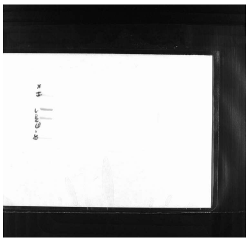
marker

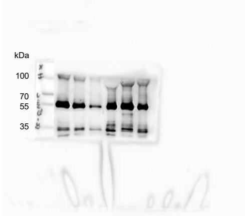

merge

γENaC

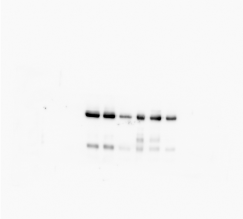
detection

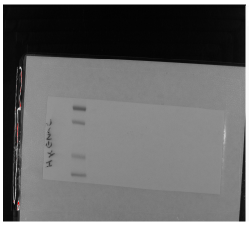
marker

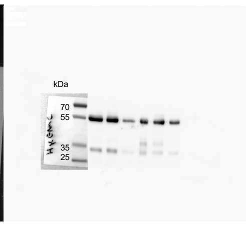

merge

CD9

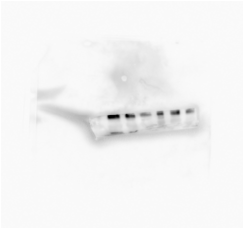
detection

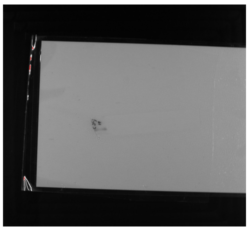
marker

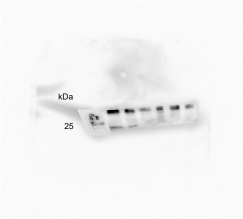

merge

NCC

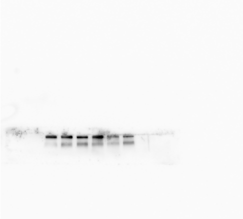
detection

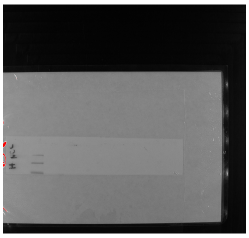
marker

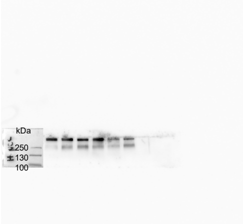

merge

NHE3

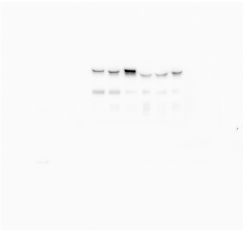
detection

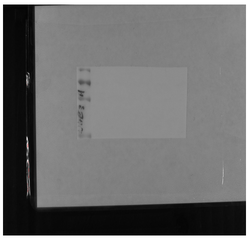
marker

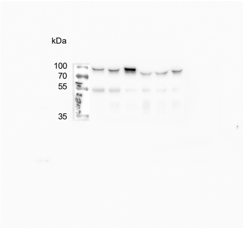

merge

NKCC2

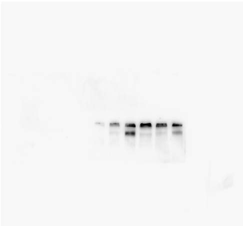
detection

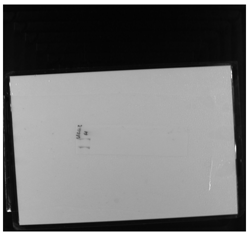
marker

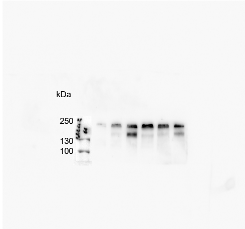

merge

Patient I

αENaC

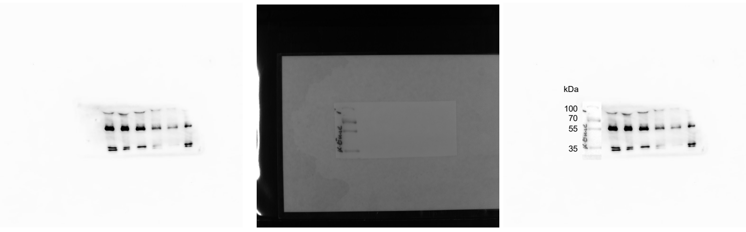

detection marker merge

CD9

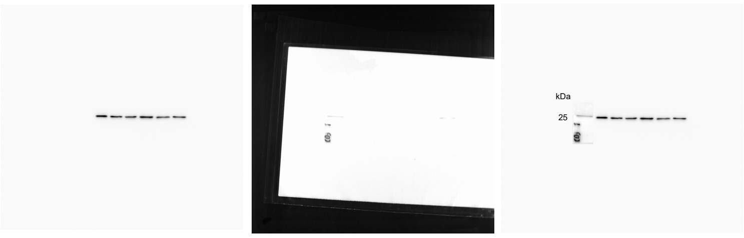

detection marker merge

NHE3

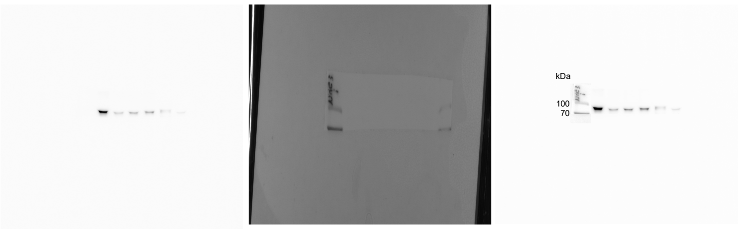

detection marker merge

NCC

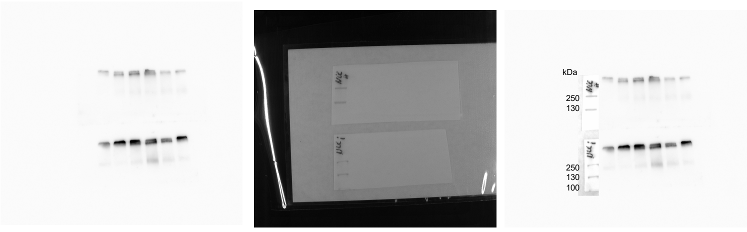

detection marker merge

Patient J

CD9

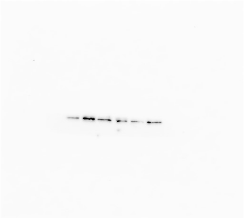

detection

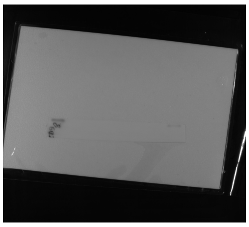

marker

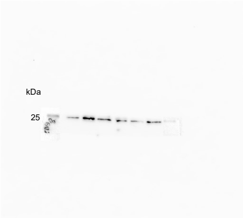

merge

NCC

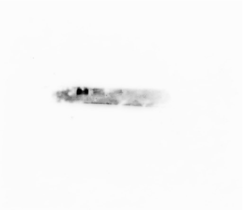

detection

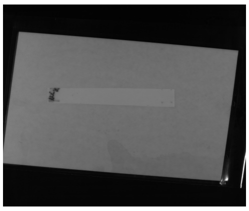

marker

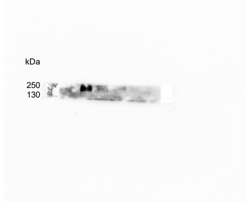

merge

NHE3

detection

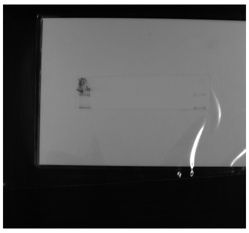

marker

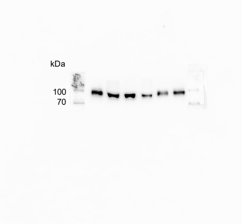

merge

NKCC2

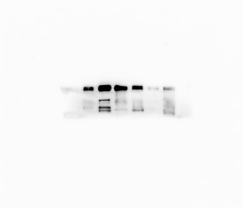

detection

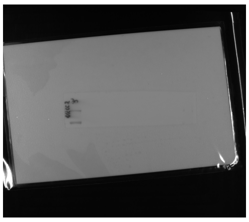

marker

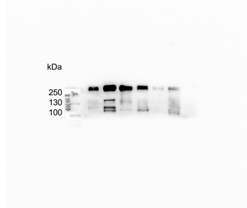

merge

αENaC

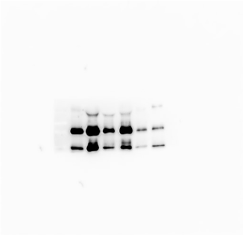

detection

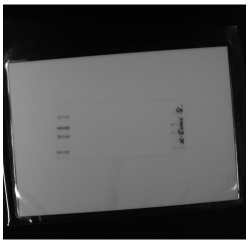

marker

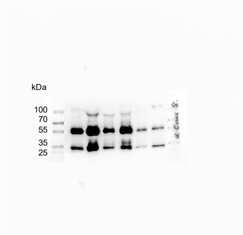

merge

Patient K

γENaC

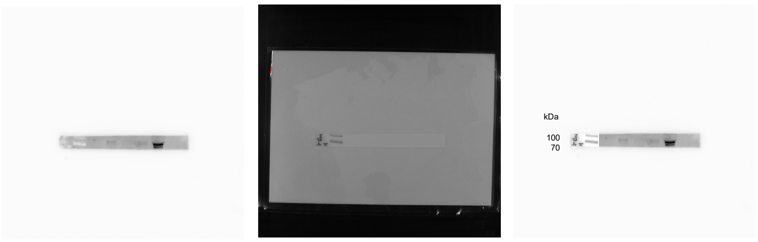

detection marker merge

CD9

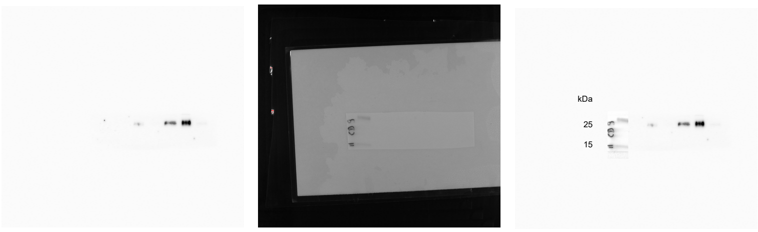

detection marker merge

Hexokinase 2

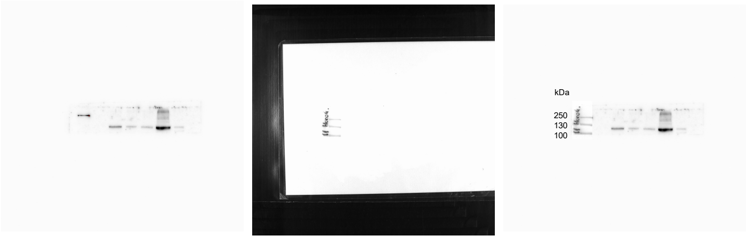

detection marker merge

Patient M

γENaC

detection

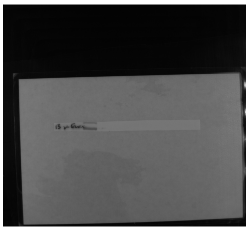
marker

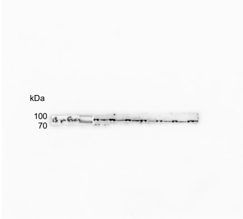

merge

CD9

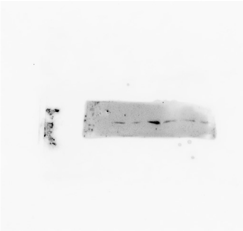
detection

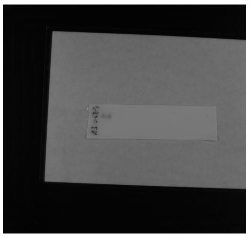
marker

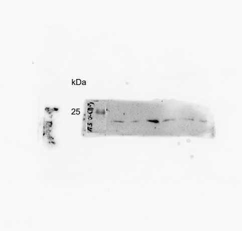

merge

Hexokinase 2

detection

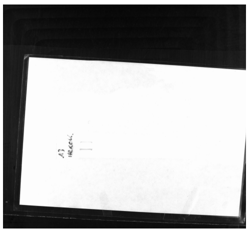
marker

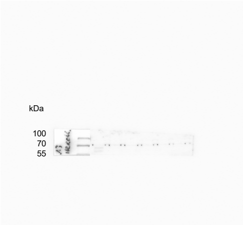

merge

GLUT1

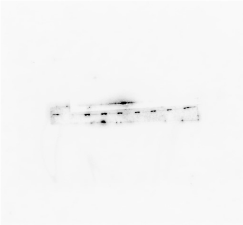
detection

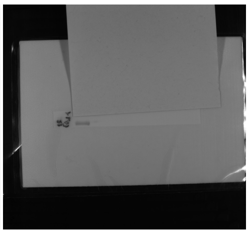
marker

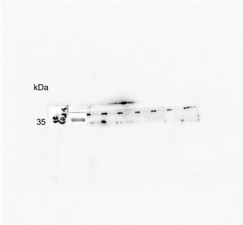

merge

Patient O

γENaC

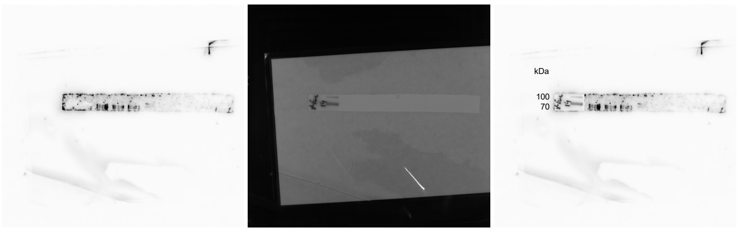

detection marker merge

CD9

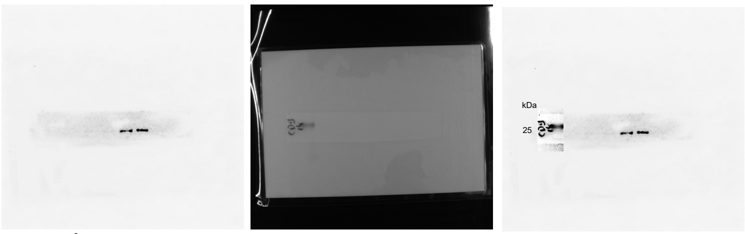

detection marker merge

NHE3

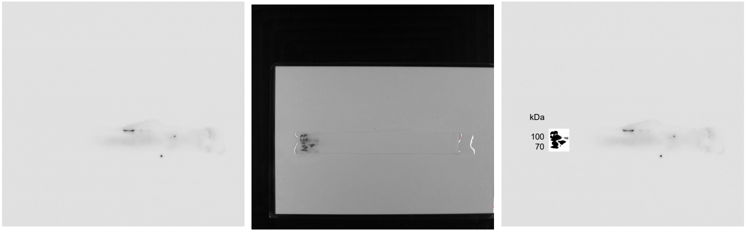

detection marker merge
